# Analysis and experimental research of transient temperature rise characteristics of high-speed cylindrical roller bearing

**DOI:** 10.1038/s41598-024-51255-9

**Published:** 2024-01-06

**Authors:** Yanfang Dong, Yafei Ma, Ming Qiu, Feifan Chen, Kai He

**Affiliations:** 1 School of Mechatronics Engineering, Henan University of Science and Technology, Luoyang, 471003 China; 2 Henan Provincial Cooperative Innovation Center for Advanced Manufacturing of Mechanical Equipment, Luoyang, 471003 China

**Keywords:** Mechanical engineering, Applied optics

## Abstract

In this paper, on one hand, the time-varying characteristics of the heat source and thermal boundary conditions of the high-speed spindle system were analyzed considering the thermal-structural coupling effect. And a transient bearing temperature field prediction method combining the thermal network method and finite element method was proposed. Furthermore, the relationships between time step, calculation efficiency and calculation results were analyzed. On the other hand, a online real-time monitoring system of the transient temperature of the cylindrical roller bearing inner ring for maximum speed of 13,000 r/min was designed and implemented using fibre optic sensing technology. Comparing with the conventional static thermal analysis results, it is verified that the simulation method proposed in this paper has higher accuracy. This paper provides a new approach for analysing and testing the thermal characteristics of high-speed spindle system.

## Introduction

The spindle system is the key unit of CNC machines. The thermal performance of the system directly affects the machining accuracy. Therefore, it is important for analyzing the thermal characteristics of the spindle system components, especially the bearings which endure the cutting force, high rotation speed and generate a lot of heat. Currently, the thermal network and finite element methods are the common thermal characteristics analysis methods for bearings. Su et al.^1,2^ designed a thermal network including the lubricating oil and contact factors, and predicted the temperature rise of motorized spindles. Liu et al.^3^ proposed a thermal resistance network model of a spindle-bearing-bearing pedestal, and investigated the thermal characteristics of the motorized spindle system. Yan et al.^4^ studied the transient characteristics of the spindle-bearing system by the thermal network coupling with the thermal-deformation. Liu et al.^5^ established a thermal resistance network model and predicted the temperature field distribution in the spindle system with reasonable accuracy. Li et al.^6^ used the thermal network method in the simulation of power output performance of thermoelectric generation on a rotating spindle. In the meantime, a large number of scholars have reported the thermal analysis of the bearing using finite element methods. Liu et al.^7^ and Li^8^ predicted thermal distribution of motorized spindles by FEM technique. Gao et al.^9^ studied the coupled vibrations of motorized spindle using FEM. Zivkovic et al.^10^ presented 3-D finite element thermal model for studying the machine tool spindle thermal characteristics.

The above research has made a comprehensive and in-depth study on the heat transfer mechanism, heat-solid coupling behavior and analysis methods of high-speed spindle bearing systems using the thermal network and finite element methods. Thermal network method analysis computes thermal solutions at the nodes; however, it cannot accurately compute the temperature field of the actual structure, particularly at the internal portion of complex objects. The finite element method solves this shortcoming by providing solutions at all locations irrespective of the size and shape of the object. However, FEM can only obtain the results under a certain operating condition without considering boundary condition dynamic changes. In view of this, considering the thermal-structural coupling effect, this paper analyzed the time-varying characteristics of the thermal boundary conditions of the high-speed spindle system in operation. A transient bearing temperature field prediction method combining the thermal network method and finite element method was proposed. The relationship between time step and computation accuracy was reported. And a online real-time monitoring system of the transient temperature of the cylindrical roller bearing inner ring for maximum speed of 13,000 r/min was designed and implemented using fibre optic sensing technology. Finally, the proposed model was verified with the test results of the online transient temperature monitoring system.

## High-speed spindle thermal dynamic characteristics analysis model

**Figure 1:**
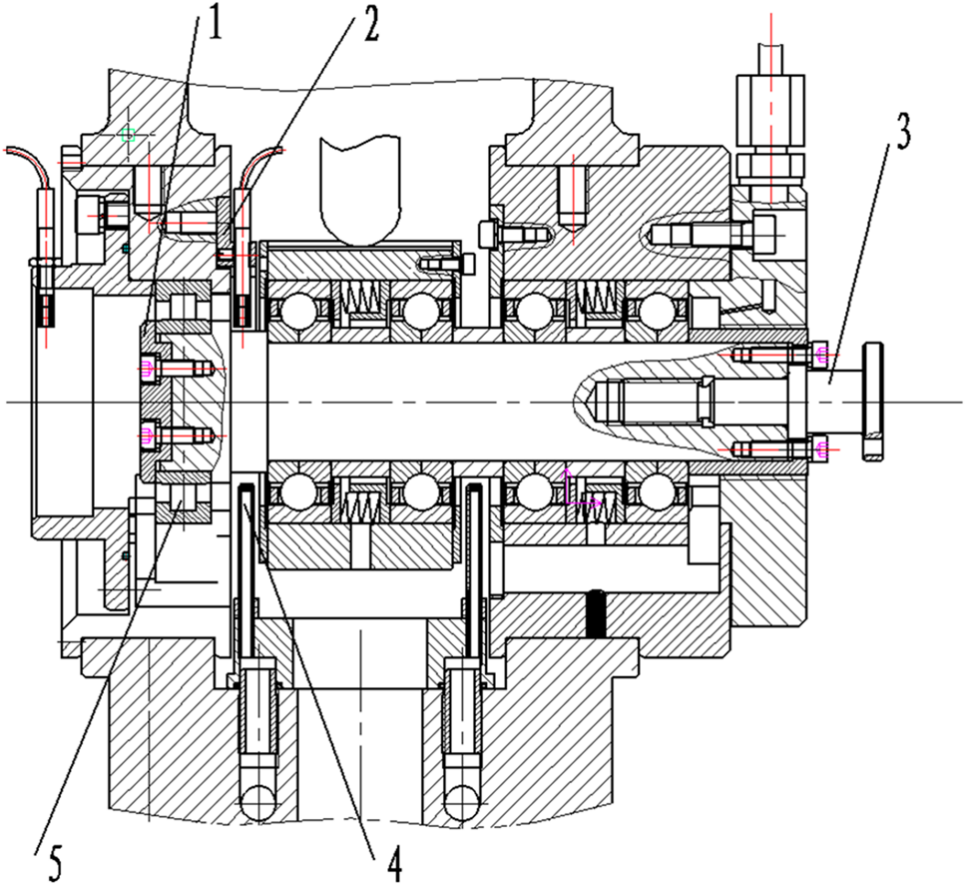
Schematic diagram of the spindle system (1. Shaft end caps 2. Sensor fixing plate 3. Coupling 4. Left oil injection nozzle 5. Test bearing).

The bearing is the main heat source of the spindle system, and the heat generation of the bearing is related to its speed, force, structure, lubrication and other conditions. The system's temperature field and thermal deformation interact through the heat source and heat dissipation boundary conditions, and finally the high-speed spindle system reach a thermal equilibrium state. The spindle system studied in this paper is shown in Fig. 1.

### Computation of loss of bearing power

The temperature rise in bearing is due to the friction between the contact surfaces during bearing rotation, including the sliding friction heat rate between the roller and the ring *Q*_*1*_, the roller churning friction heat rate *Q*_*2*_, the sliding friction heat rate between the cage and the ring surface *Q*_*3*_, the sliding friction heat rate between the roller and the cage pocket *Q*_*4*_, the elastic hysteresis loss heat rate *Q*_*5*_, rolling and the sliding friction heat rate *Q*_*6*_ between the roller end face and the retaining edge. The frictional heat rate between each part is calculated based on the elastic flow lubrication contact, and the detailed process can be referred to the literature^11^, so the total heat rate of the bearing *Q* is as follows:

1 $$ Q = \sum\limits_{i = 1}^{6} {Q_{i} } $$

**Figure 2:**
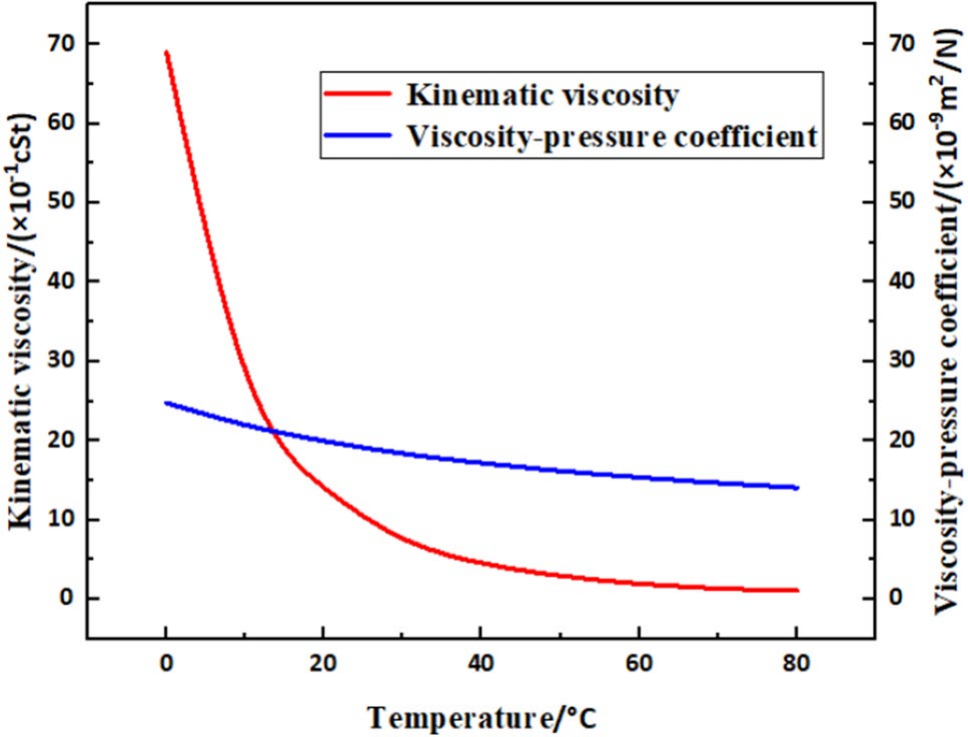
Variation of lubricant physical parameters with respect to temperature.

The viscosity-temperature characteristics of the lubricant have a considerable effect on the heat generation. Therefore, it was considered during subsequent iterations of the calculation. And the physical parameters of the No. 46 lubricant used in this study are shown in Fig. 2.

### Identifying thermal nodes and boundary parameters

**Figure 3:**
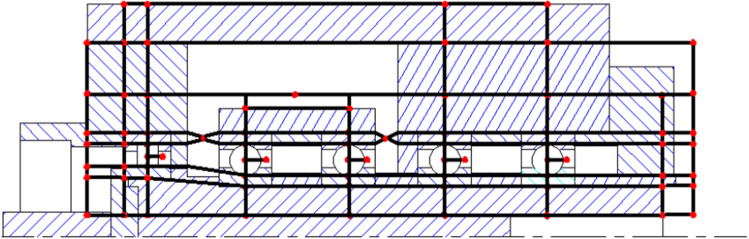
Schematic representation of test spindle structure and identification of thermal node arrangements.

The circumferential heat transfer was not considered as the system symmetry along the centre line of the spindle. The thermal nodes are divided according to the test spindle structure and the material characteristics of each portion, as shown in Fig. 3.

The effect of thermal radiation can be ignored as the study was performed below 200 °C. The heat conduction and thermal convection was considered. The formulation of considered thermal resistances has been given in Table 1^11^.

**Table 1:** The thermal resistance calculation formula.

Type	Thermal resistance (K/W)
Radial conduction thermal resistance	$R_{r} = \frac{{\ln \left( {d_{e} /d_{i} } \right)}}{{2{\uppi }kL_{c} }}$
Roller radial conduction thermal resistance	$R_{ror} = \frac{1}{{{\uppi }kL_{c} }}$
Axial conduction thermal resistance	$R_{a} = \frac{{L_{c} }}{kS}$
Thermal resistance of roller and raceway contact conduction	$R_{c} = \frac{1}{{\uppi }}\left( \frac{a}{b} \right)\frac{1}{{ka\sqrt {Pe} }}$
Convective thermal resistance	$R_{h} = \frac{1}{{Ah_{v} }}$

*k* is the thermal conductivity of the corresponding material, *L*_*c*_ denotes the characteristic length, *d*_*e*_ and *d*_*i*_ represent the inner and outer diameters of the circular structure, *S* signifies the cross-sectional area, *a* and *b* denote the long and short semi-axes of the contact ellipse, *Pe* represents the Peclet number, *A* and *h*_*v*_ signifies the convective heat transfer area and coefficient. These variables were computed for different flow states. The contact thermal resistance of the shaft and inner ring was 0.02 m^2^ K/W and it was 0.1 m^2^ K/W between the outer ring and bearing housing^12^.

### Formulating transient heat balance equation

The thermal network calculated the transient temperature considering the heat flow at node i equal to the increase in energy within a specific volume^13^.

2 $$ q_{i} = \rho_{i} C_{\rho i} V_{i} \frac{{dT_{i} }}{dt} $$

where, *qi*, *ρi*, *Cρi*, *Vi* and *Ti* represent the heat flow, material density, specific heat, corresponding volume and temperature at the i-th node, respectively, *dTi/dt* is the rate of temperature rise at the i-th node.

For example, assuming that heat *Q*_*0*_ from node 0 flows into neighbouring nodes 1, 2, 3 and 4, the transient heat balance equation at that location can be expressed as

3 $$ Q_{0} - C_{0} \rho_{0} V_{0} \frac{{dT_{0} }}{{t_{0} }} = \sum\limits_{i = 1}^{4} {\frac{{T_{0} - T_{i} }}{{R_{0 - i} }}} $$

where, T_i_ is the transient temperature of node *i*, and *R*_*0−i*_ is the thermal resistance between the neighbouring nodes.

**Figure 4:**
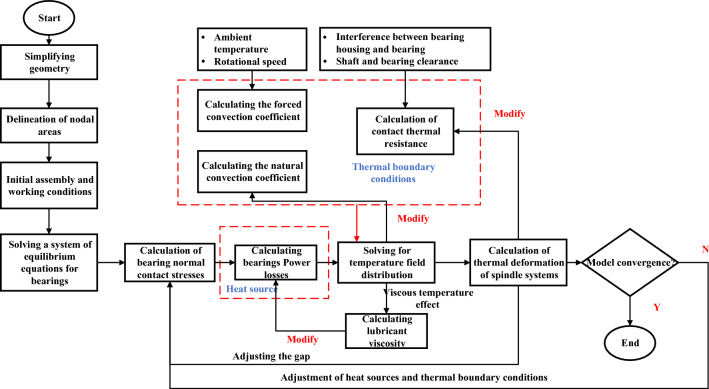
The flowchart for computing the high-speed spindle thermal structure coupling process.

**Figure 5:**
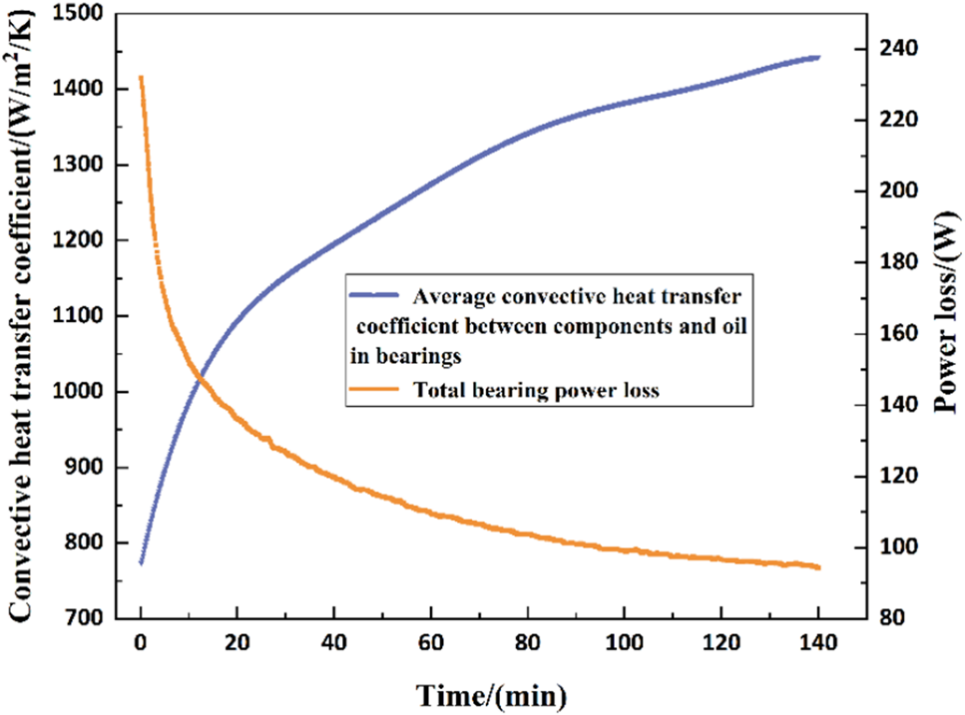
Variation of thermal parameter transient characteristic with respect to time.

Based on the above analysis, the general procedure for solving the transient temperature field of the spindle system based on thermal network method is presented in Fig. 4^14^. And the convergence criterion of this model is: until the temperature difference between the bearing balls at two adjacent time points is less than 0.1 ℃, the closed-loop iterative modelling is terminated, and then the results are outputted when the model of the thermal-structural interaction properties is converged. Under a radial force of 400 N and speed of 9000 r/min (used for all subsequent analyses), the transient characteristics of the thermal parameters are shown in Fig. 5.

## Transient thermal simulation and analysis

### Finite element modeling of the spindle system

**Figure 6:**
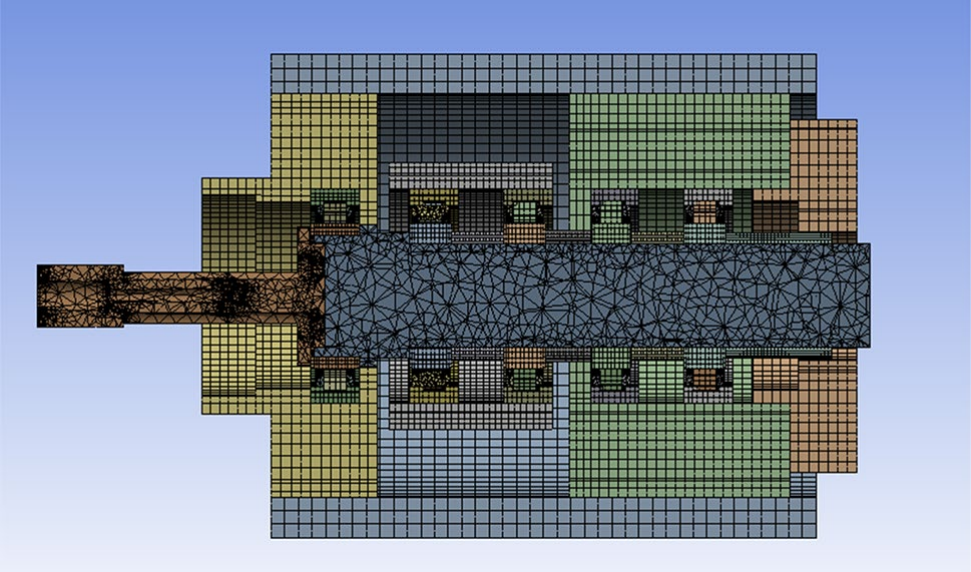
Finite element model of the spindle bearing system.

Considering the time cost of the calculation, the 3D model needs to be simplified by ignoring secondary structures, such as springs and nuts which have little influence on the heat transfer of the system. In addition, to ensure the calculation accuracy, the mesh grid of the bearing area is to be refined. The results of the mesh division are shown in Fig. 6, with total of 277,328 cells, 1195,256 nodes and an average mesh mass of 0.9. The material properties of the system components are shown in Table 2.

**Table 2:** System material parameters.

Parameter	Parameter value		
Material	40Cr (sleeve)	9Cr18 (end caps, shaft)	Cu (substrates)
Density (g/cm^3^)	7.85	7.7	8.94
Thermal conductivity (W/m K)	60.5	29.3	398
Poisson's ratio	0.29	0.3	0.34
Modulus of elasticity (GPa)	211	200	110
Coefficient of thermal expansion (10^−6^/℃)	11	10.5	17.5

### Thermal boundary conditions and thermal load

The thermal boundary conditions are set as follows.

1. Loading of heat generation in the form of Heat Flow onto the roller, inner and outer raceway surfaces.
2. Natural air convection was applied to the housing and stationary surfaces. Forced air convection was applied to the rotating surfaces. Forced convection of lubricant was applied to the inner and outer raceway, roller surfaces. Temperature Dependent was selected as the setting method.

Meanwhile, a novel aspect of this paper is that the dynamic variation of the thermal parameters is considered. Therefore, choosing an optimal fitting function for thermal loading is required. Taking the dynamic power loss in Fig. 5, the comparison of the different curve fitting methods was made, as given in Table 3.

**Table 3:** Comparison of fit results.

Evaluation metrics	Curve fitting method		
	Fourier (8th order)	Gaussian (8th order)	Polynomial (9th order)
SSE	87.48	44.23	864.6
R2	0.9998	0.9999	0.9981
Adjusted R2	0.9998	0.9999	0.9981
RMSE	0.3262	0.2328	1.021

The goodness of fit is inversely proportional to the sum of squared errors SSE and root mean square error RMSE, directly associated with the coefficient of determination R2 and the corrected coefficient of determination Adjusted R2. According to the results of the comparison, the 8th-order Gaussian fitting function as Eq. (4) is chosen to simulate the dynamic changes of the heat source. The parameters in the expression are given in Table 4.

4 $$ \begin{gathered} f\left( t \right) = a_{1} \cdot \exp \left( { - \left( {\left( {t - b_{1} } \right)/c_{1} } \right)^{2} } \right) + a_{2} \cdot \exp \left( { - \left( {\left( {t - b_{2} } \right)/c_{2} } \right)^{2} } \right) + \hfill \\ a_{3} \cdot \exp \left( { - \left( {\left( {t - b_{3} } \right)/c_{3} } \right)^{2} } \right) + a_{4} \cdot \exp \left( { - \left( {\left( {t - b_{4} } \right)/c_{4} } \right)^{2} } \right) + \hfill \\ a_{5} \cdot \exp \left( { - \left( {\left( {t - b_{5} } \right)/c_{5} } \right)^{2} } \right) + a_{6} \cdot \exp \left( { - \left( {\left( {t - b_{6} } \right)/c_{6} } \right)^{2} } \right) + \hfill \\ a_{7} \cdot \exp \left( { - \left( {\left( {t - b_{7} } \right)/c_{7} } \right)^{2} } \right) + a_{8} \cdot \exp \left( { - \left( {\left( {t - b_{8} } \right)/c_{8} } \right)^{2} } \right) \hfill \\ \end{gathered} $$

**Table 4:** Values of fitting parameters for Eq. (4).

*i*	*ai*	*bi*	*ci*
1	43.88	− 0.8531	3.107
2	1.6 × 1013	− 475	92.5
3	− 0.04238	29.03	15.77
4	− 0.7573	40.73	6.173
5	64.79	− 20.24	71.87
6	0.5006	83.4	3.279
7	0.1944	91.96	3.245
8	95.52	111.7	243.5

### Transient temperature field analysis

**Figure 7:**
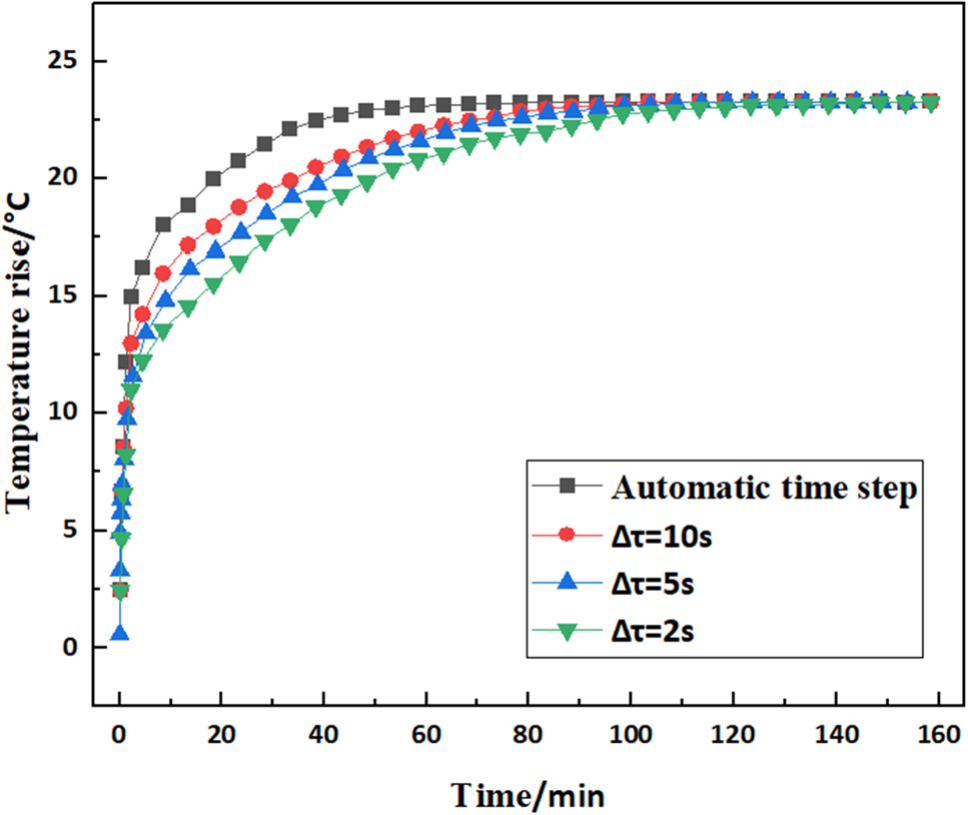
Effect of iterative step size on transient temperature rise of the surface of the outer ring.

Firstly, the effect of iteration step on temperature rise is analyzed. The transient temperature rise of the bearing outer ring surface with respect to iteration step under ambient temperature of 19.5 ℃ is shown in Fig. 7.

As can be seen from Fig. 7, the larger the iteration step of the simulation, the faster the temperature rise curve converges (the automatic time step of the program control is almost always greater than 50 s), and the faster the system reaches thermal equilibrium. The iteration step only affects the initial convergence rate of the temperature rise curve and does not affect the steady-state temperature value. This is due to smaller step sizes need more accurate modelling of the system dynamics. However, smaller step sizes lead to an increase in the time cost of the algorithm execution, as more iterative steps need to be performed to reach the desired time point. Conversely, larger step sizes may fail to capture the details of the system's dynamic changes, leading to compromised numerical accuracy. Therefore, the actual analysis process needs to be combined with the actual to set the iterative step size.

**Figure 8:**
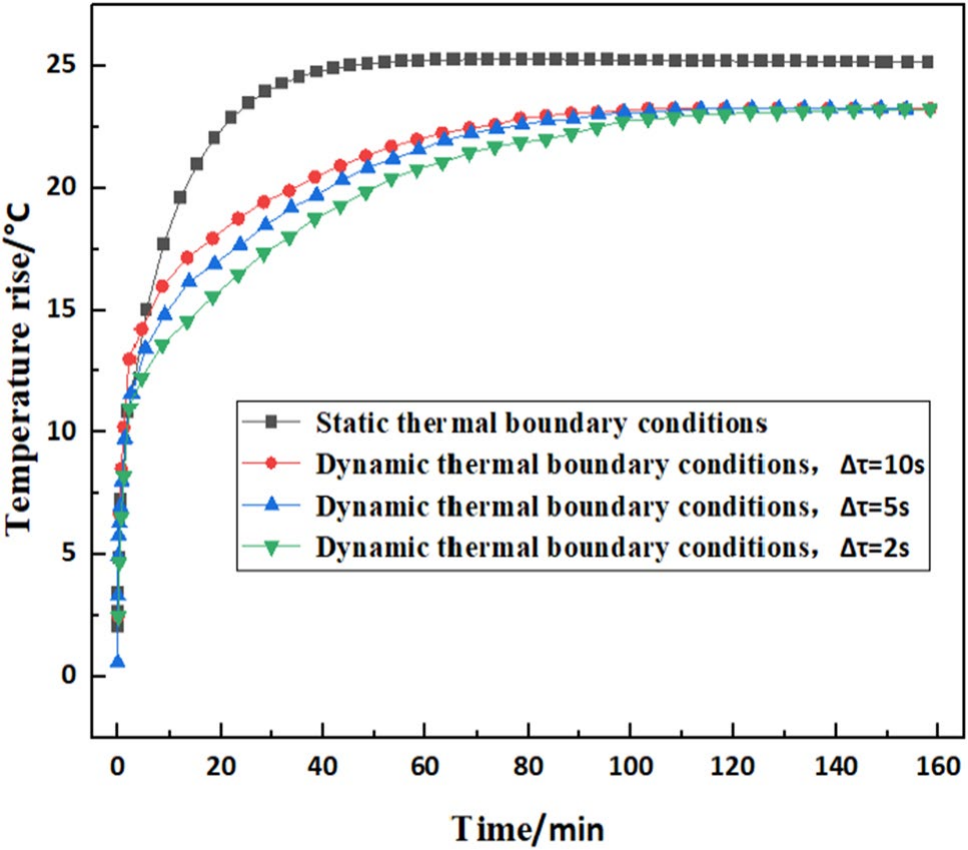
Comparison of static and dynamic thermal parameter simulation results.

Secondly, the comparison of temperature rise under dynamic and static thermal boundary conditions has been done. Figure 8 showed the variation of temperature rise with static and dynamic boundary conditions at different time steps. At the initial stage, the trend of temperature rise under the two boundaries is consistent. However, the final values results were significantly higher. This was due to the static thermal parameters themselves that did not contain the thermal-structural coupling effects of the system. For example, the viscosity of the lubricant decreases significantly decreases with the increase of temperature, and the bearing power loss is variable with time. Therefore, the constant power losses and heat transfer coefficients calculated under the initial operating conditions were substituted. It also shows that the temperature prediction method proposed in this paper will be more accurate than the static boundary. On the basis of the above analysis, the simulation of bearing temperature field has been completed.

## Test verification

### Test platform

**Figure 9:**
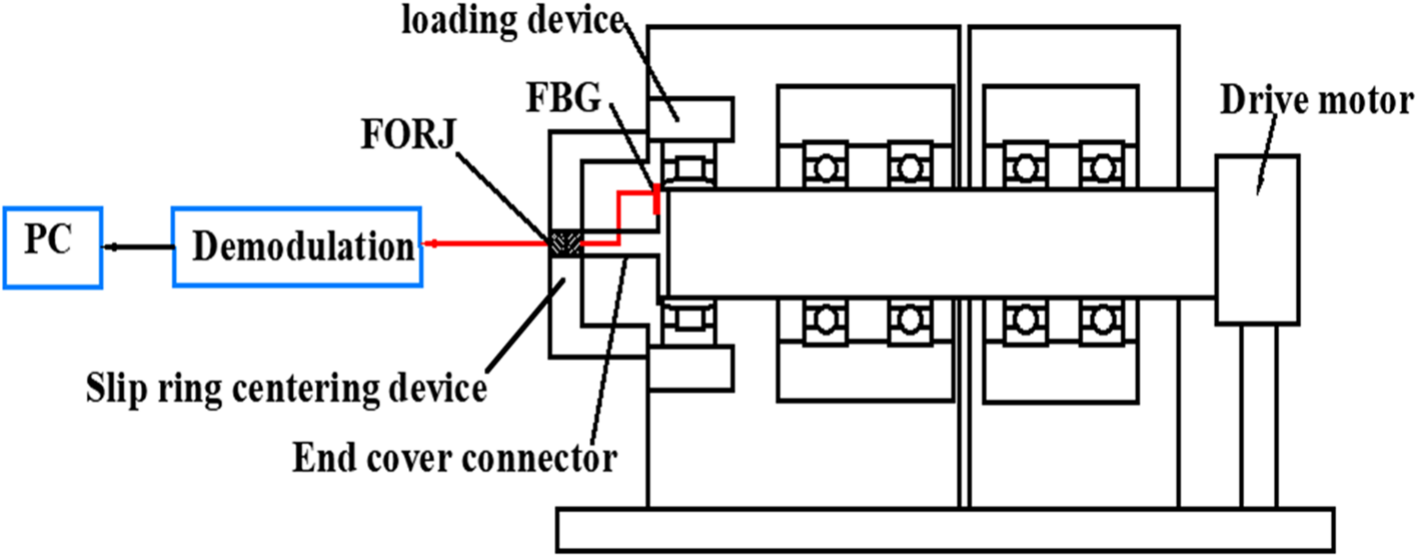
Schematic diagram of the temperature test system for bearing inner ring based on FBG.

The temperature rise test platform was required to verify the accuracy of the previously described model and the proposed simulation method. According to the structure of the spindle system used in this study, a bearing inner ring real-time temperature online monitoring system based on fibre optic sensing and rotary connector (FORJ) was designed (Fig. 9).

### Fibre sensing principle and FBG calibration

The center wavelength drift of the fibre grating can be expressed as:

5 $$ \frac{{\Delta \lambda_{B} }}{{\lambda_{B} }} = K_{T} \cdot \Delta T + K_{\varepsilon } \cdot \varepsilon $$

where, *ΔT* and ε denotes the temperature and strain change. Equation (5) is derived without considering the cross-sensitivity. The temperature sensitivity coefficient is *K*_*T*_ = 7.22 × 10–6/°C, and the strain sensitivity coefficient is *K*_*ε*_ = 0.784.

**Figure 10:**
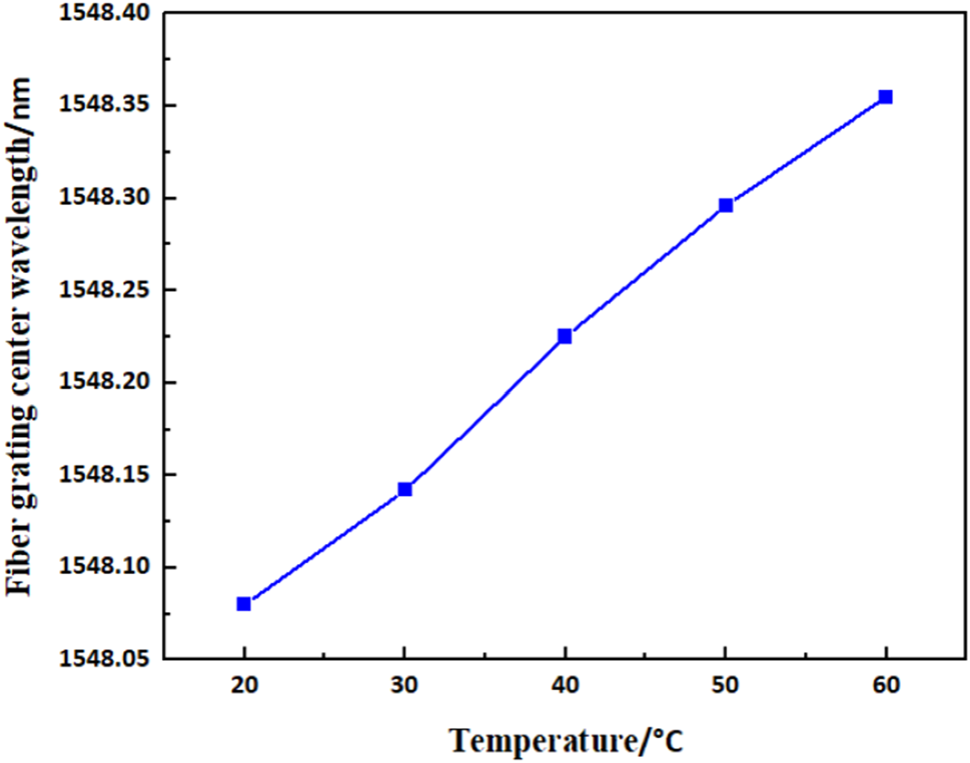
Calibration results of fibre Bragg grating sensors.

To verify the accuracy of temperature measurement, the FBG was placed in the temperature control box for calibrating, and the results are shown in Fig. 10. There was a good linear relationship between the centre wavelength change of the FBG and the temperature rise, which proves FBG can perform the measurement of the bearing temperature rise.

### FBG package structure design

**Figure 11:**
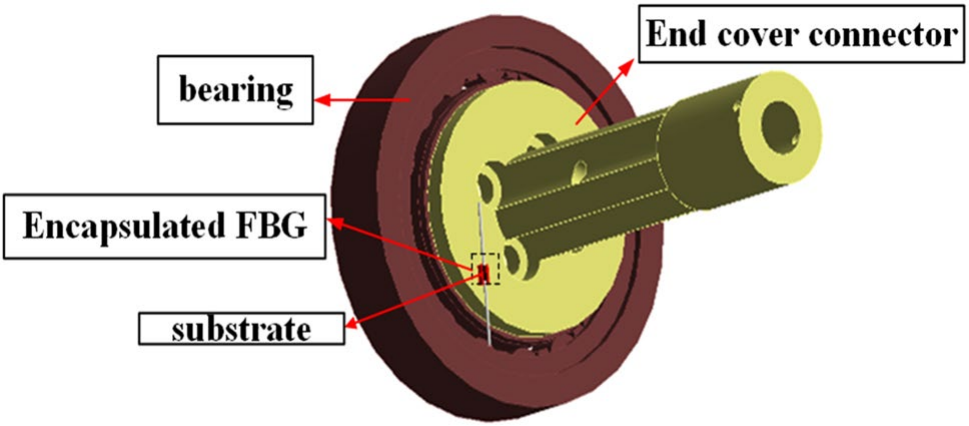
Diagram of FBG package.

Fibre Bragg grating is sensitive to strain and temperature at the same time. For obtaining accurate temperature rise of the bearing inner ring, it was necessary to avoid the effect of strain and the environmental influence, so the packaging method shown in Fig. 11 was designed.

**Figure 12:**
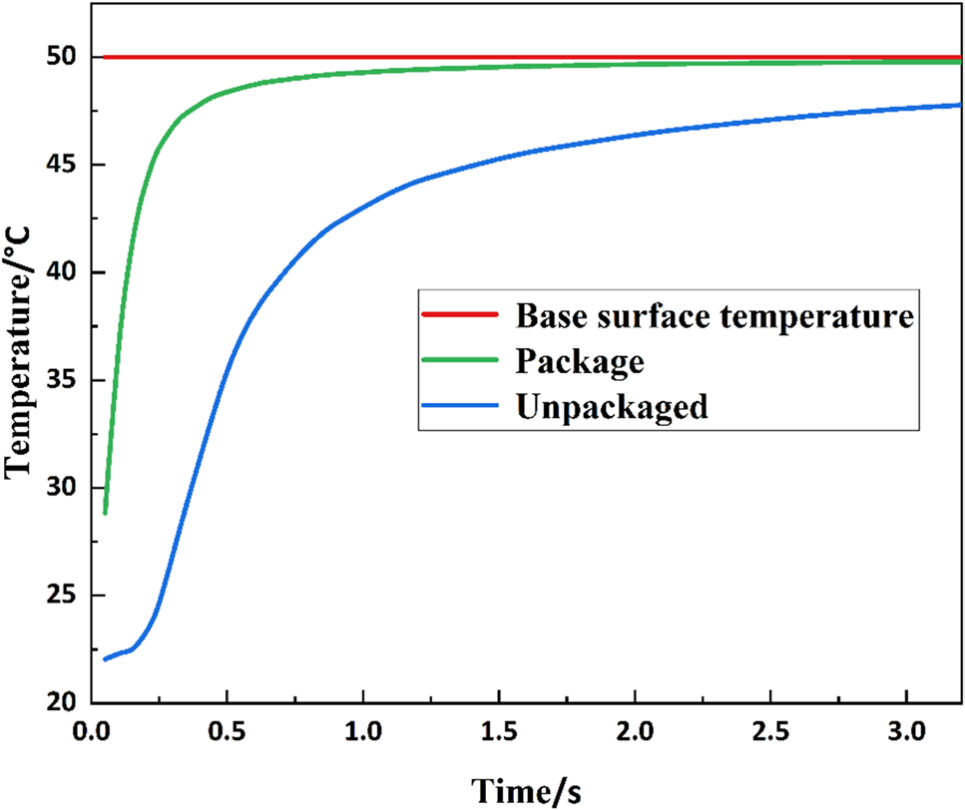
Test result comparison between the packaged and unpackaged FBG sensor.

The grating was placed on a thin-walled copper substrate with a groove (red part in Fig. 11), and the thermally conductive silicone grease was injected into the groove to fix the substrate during encapsulation. This structure can reduce the effect of the groove gap on heat transfer and use the silicone grease's viscosity to reduce the fibre's micro-strain due to centrifugation. The test result comparison between the packaged and unpackaged FBG sensor has also been done through the thermal model. The contact interface between the connector and the bearing inner ring end face were set as a constant temperature, and then the temperature change of the FBG surface was monitored. The simulation results are shown in Fig. 12.

It is evident in Fig. 12 that the timeliness and accuracy of the packaged FBG temperature measurement are better than the unpackaged one.

**Figure 13:**
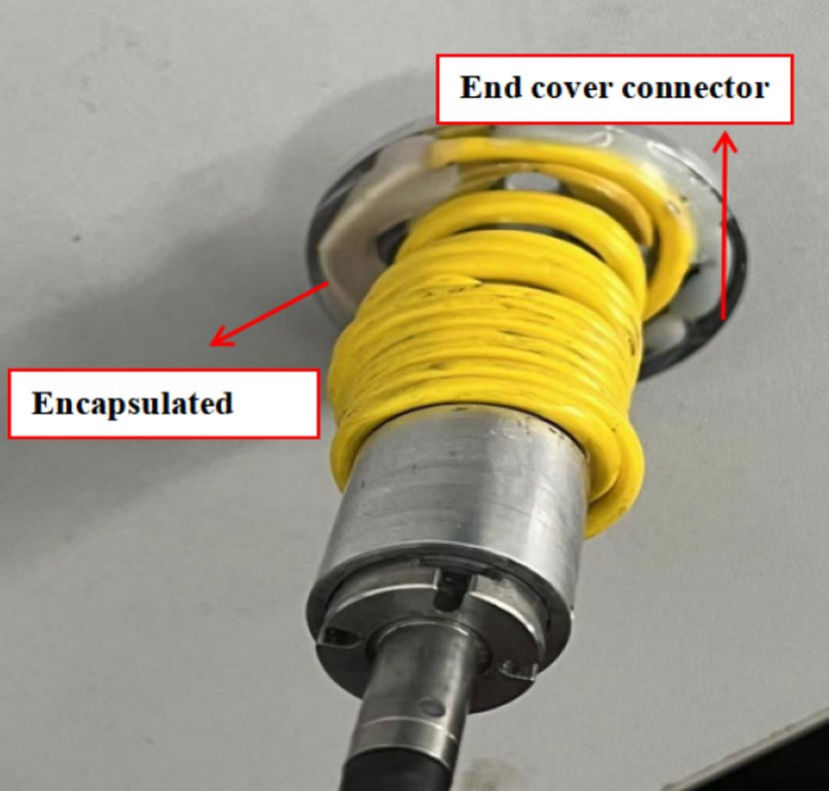
End cap connector and FBG sensor.

In addition, to prevent the influence of oil mist and high temperature, the substrate is fixed with the C-3 high temperature resistant inorganic adhesive, as shown in Fig. 13. Noting that the end of the fibre should be kept free when fixing the outer cladding, to prevent the influence of stretching caused by the substrate thermal expansion.

After the above treatment, the FBG was used almost strain-free, so that the relative wavelength drift under the effect of temperature can be expressed as given in Eq. (6):

6 $$ \frac{{\Delta \lambda_{B} }}{{\lambda_{B} }} = \left[ {\alpha + \xi + \left( {1 - P_{\varepsilon } } \right)\left( {\alpha + \alpha_{s} } \right)} \right]\Delta T $$

where, *α* and *ξ* are the thermal expansion coefficient and thermo-optical coefficient of the fibre core, respectively. *Pε* denotes the effective elasticity coefficient, and *α*_*s*_ represents the thermal expansion coefficient of the substrate material.

### Test results and comparative analysis

**Figure 14:**
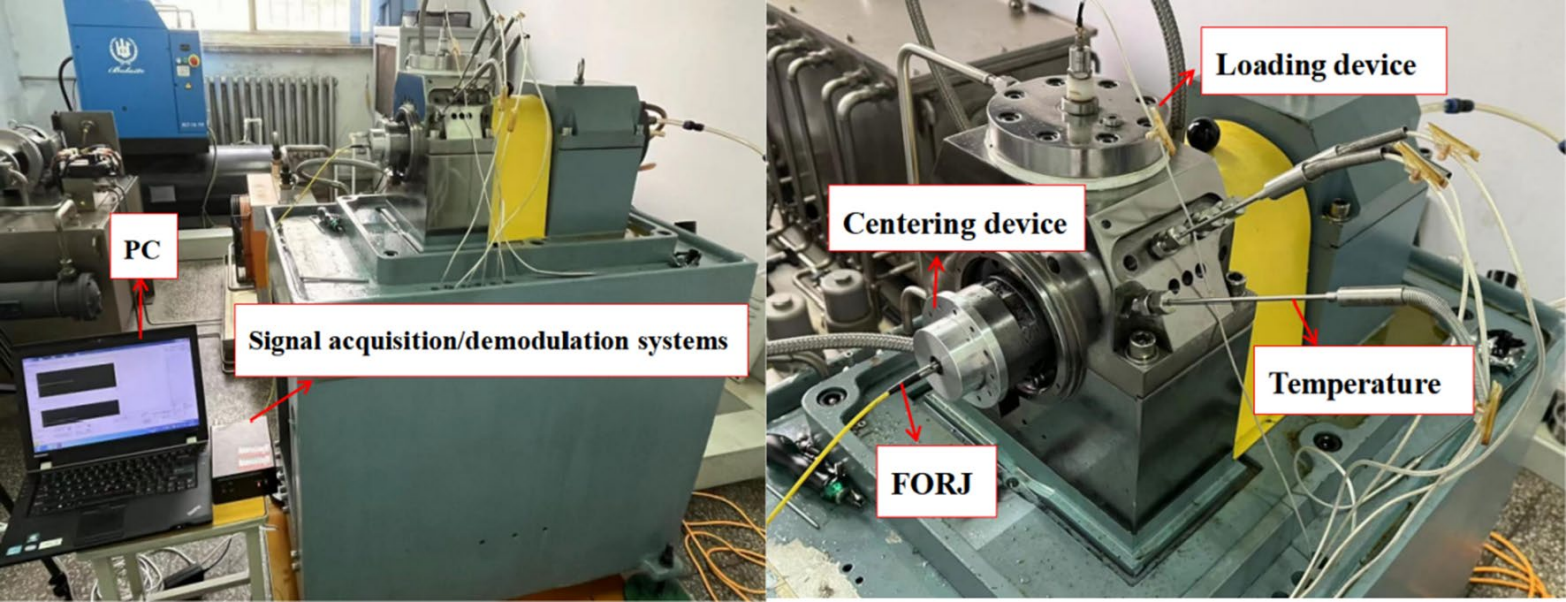
Experimental setup.

**Figure 15:**
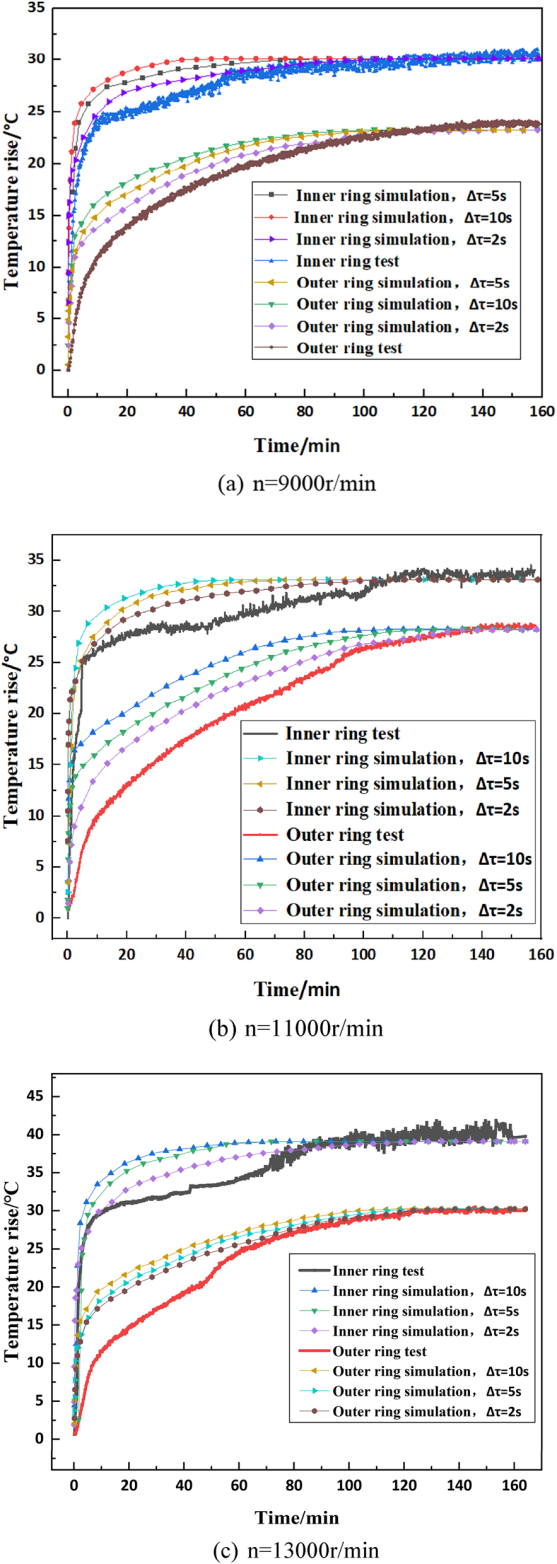
Comparison of results under different conditions.

The test platform and sensor arrangement are shown in Fig. 14. The specifications, test conditions and other parameters of the spindle system and bearings are shown in Table 5. The results of the simulation and experimental tests under different speeds are shown in Fig. 15.

**Table 5:** Technical specifications, test conditions and other parameters of the test stand.

Spindle type	Mechanical spindle
Drive mode	Electrospindle drive
Lubrication method	Oil injection (room temperature ~ + 200 ℃)
Test bearing model	NU1007
Sensor	Outer ring: thermocouple temperature sensor Inner ring: fibre grating sensor
Measurement and control mode	Outer circle: PC automatic monitoring and recording Inner circle: optical signal acquisition instrument and PC
Ambient temperature (°C)	19.5
Measured speed (r min^−1^)	9000, 11,000, 13,000
Test load	Radial 400N

The results of the simulation and experimental tests at different speeds are shown in Fig. 15.

It can be seen that the method proposed in this paper has sufficient accuracy to simulate the temperature rise characteristics of the spindle bearing system, and the smaller the iteration step, the higher the simulation accuracy. With the decrease of iteration step, the simulation temperature rise is closer to the real temperature rise in the whole process.

In addition, reducing the iteration step length can get better simulation results. In this paper, the step length was reduced from Δτ = 10 s to Δτ = 5 s, but the computation times is increased by three times. In the meantime, it can also be seen that the iteration step has no effect on the final thermal balance temperature of the bearing, so the iteration step length should be chosen reasonably in practical engineering.

## Conclusion

This paper comprehensively considers the thermal-structural coupling effect, analyses the time-varying characteristics of the thermal parameter conditions of the high-speed spindle system under the operating condition, combines the thermal network method with the finite element method for the prediction of the transient temperature field of the bearings, and designs a high speed bearing inner and outer ring temperature measurement test system for verification. The main conclusions are shown as follows.

1. A method for predicting the transient temperature field of bearings by combining the thermal network method with the finite element method is proposed, and it is demonstrated experimentally that the method has higher accuracy than the simulation using static thermal boundary conditions.
2. Online monitoring of the inner ring transient temperature of cylindrical roller bearings at high speed (maximum test speed 13,000 r/min) is designed using fibre optic rotary connectors and FBG sensors.
3. Shortening the iteration step can improve the simulation accuracy in the ramp-up phase using dynamic thermal boundary condition.

Through the study of this paper, it is evident that the bearing temperature prediction will be more accurate by using the dynamic thermal boundary, and the changes in boundary conditions cannot be ignored. Simultaneously, the designed online temperature monitoring system of bearing inner ring can be extended to the dynamic strain test of high speed rotating parts, such as gear, aero-engine blades.

## Data Availability

The datasets used and analyzed during the current study available from the corresponding author on reasonable request.
